# The Dose-Effect of Icariin on the Proliferation and Osteogenic Differentiation of Human Bone Mesenchymal Stem Cells

**DOI:** 10.3390/molecules161210123

**Published:** 2011-12-06

**Authors:** Jun-Jun Fan, Liang-Guo Cao, Tao Wu, De-Xin Wang, Dan Jin, Shan Jiang, Zhi-Yong Zhang, Long Bi, Guo-Xian Pei

**Affiliations:** 1 Department of Orthopaedic Surgery, Xi Jing Hospital, Fourth Military Medical University, # 88 Jiefang Road, Xi’an 710032, China; 2 Department of Orthopaedic Surgery, Southern Hospital, Southern Medical University, # 1838 North Guangzhou Road, Guangzhou 510515, China

**Keywords:** icariin, dose-effect, human bone mesenchymal stem cells, osteogenic differentiation, proliferation

## Abstract

Icariin had been reported as a potential agent for osteogenesis, but the dose-effect relationship needed further research to realize the clinical application of icariin. We isolated and purified human bone mesenchymal stem cells (hBMSCs) and stimulated them with different concentrations of icariin. The cytotoxicity of icariin was evaluated by the methylthiazolytetrazolium (MTT) assay method. The proliferation and osteogenic differentiation of such hBMSCs were investigated for different concentrations of icariin. We found that icariin had a dose-dependent effect on the proliferation and osteogenic differentiation of hBMSCs in a suitable concentration range from 10^−9^ M to 10^−6^ M, but at concentrations above 10^−5^ M, the cytotoxicity limited its use. The extremely low cost of icariin and its high abundance make it appealing for bone regeneration.

## 1. Introduction

The treatment of serious osseous defects remains a great challenge in orthopedic surgery [1]. With the introduction of tissue engineering, scaffolds holding the *in vitro* expanded seed cells are now widely considered a potential alternative to autogenous bone grafting and show a most promising future [2]. Because bone marrow-derived mesenchymal stem cells (BMSCs) have the potential for multiple differentiations and can differentiate to osteoblasts by the induction of growth factors (GFs), they have become the most commonly applied seed cells in bone tissue engineering and clinical practice [3].

To enhance and guarantee the differentiation of seed cells into functional bone matrix-producing cells, GFs and/or cytokines administered by direct protein delivery or viral gene delivery are necessary. Numerous GFs, such as bone morphogenetic proteins (BMPs), platelet-derived growth factor (PDGF), transforming growth factor-β (TGF-β), and insulin-like growth factors (IGFs), have been extensively studied and showed promisingly positive effects on the proliferation or/and differentiation of seed cells [4,5]. However, the high cost and rapid degradation of such expensive GFs limit their widespread use, especially in clinics [6]. Therefore, there is an urgent need to develop some alternative osteogenic products or drugs with higher efficacies and lower costs than GFs [7].

Some medical herbals have been widely used in the treatment of fractures and bone disorders for thousands of years in Asia [8,9]. *Epimedii herba* is one of the most frequently used herbs in formulas that are prescribed for the treatment of osteoporosis in China, Japan and Korea [9]. Additionally, *Epimedii herba* has been recorded in the Chinese Pharmacopoeia (2000 and 2005 editions) as a traditional Chinese medicine. In many studies, *Epimedii herba* showed a therapeutic effect on osteoporosis animal models [8,9]. Icariin also showed positive effects on the proliferation and osteogenic differentiation in many recent studies, and the mechanism by which icariin enhances the proliferation and differentiation was mainly through BMP-2 and Cbfa1 expression [10,11,12], but its dose-effect relationship needed further research to realize the clinical application of icariin. Thus we isolated and purified hBMSCs from healthy volunteers. Then, hBMSCs at the third passage were cultured with different concentrations of icariin. The cytotoxicity, proliferation and osteogenic differentiation of such hBMSCs were then investigated and compared.

## 2. Results and Discussion

### 2.1. Cells Observation

**Figure 1:**
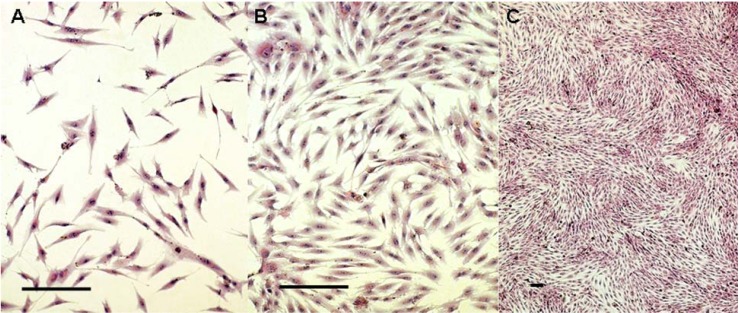
Morphological observationof the hBMSCs at 1 (Figure 1A), 5 (Figure 1B) and 10 (Figure 1C) days later. (Hematoxylin and Eosin staining. Bars indicate 100 μm)

By 4 to 8 hours after passage, the cells were adherent. Twenty-four hours later, the adherent cells showed a fusiform shape with no evidence of mitosis (Figure 1A). Three to five days later, the cells increased and showed directionality in their arrangements (Figure 1B). After 10 to 12 days, the adherent cells propagated into whirlpool-like confluence (Figure 1C).

### 2.2. Identification of BMSCs

**Figure 2:**
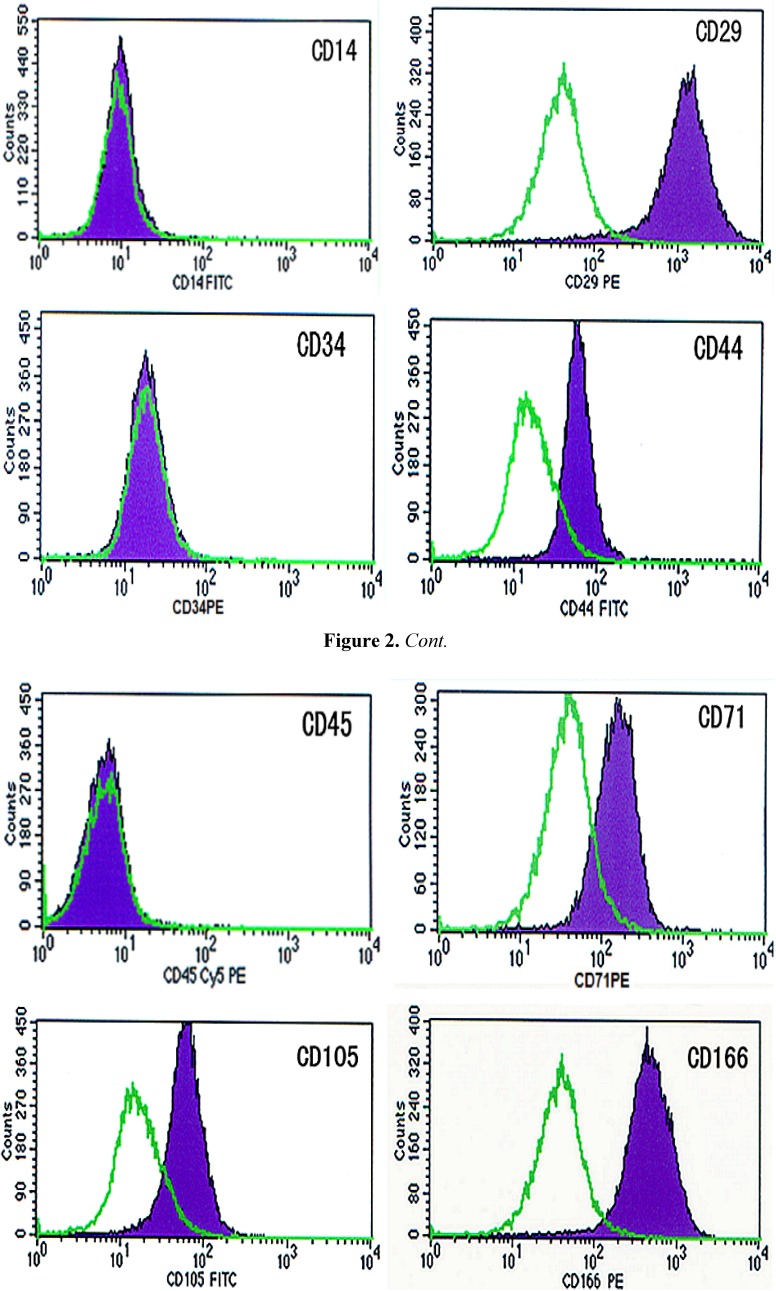
The immunophenotype of cells.

The immunophenotype of cells at the second passage was investigated via quantitative flow cytometry. All cells were highly positive for the surface antigens CD29 (93.8%), CD44 (85.98%), CD71 (72.19%), CD105 (79.28%) and CD166 (97.42%). Moreover, expression of the surface molecules CD14 (0.95%), CD34 (1.45%) and CD45 (0.73%) were below the detection limit (Figure 2).

### 2.3. hBMSC Differentiation Assay

**Figure 3:**
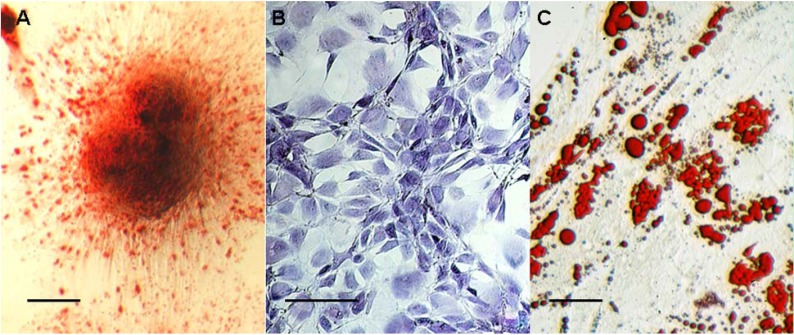
The hBMSC differentiation assay by alizarin red (Figure 3A), toludine blue (Figure 3B)and lipid droplets (Figure 3C) (bars indicate 100 μm).

Osteogenic differentiation showed that the wells were almost completely covered with mineralized deposits, as revealed using alizarin red staining after 21 days (Figure 3A). Under chondrogenic culture conditions, 14 days later, clones of cells showed positive staining of cartilage matrix using toluidine blue staining (Figure 3B). With regard to adipogenic differentiation, cells showed a larger number of clusters of lipid droplets after 21 days of adipogenic differentiation (Figure 3C).

### 2.4. Cytotoxicity of Icariin

**Figure 4:**
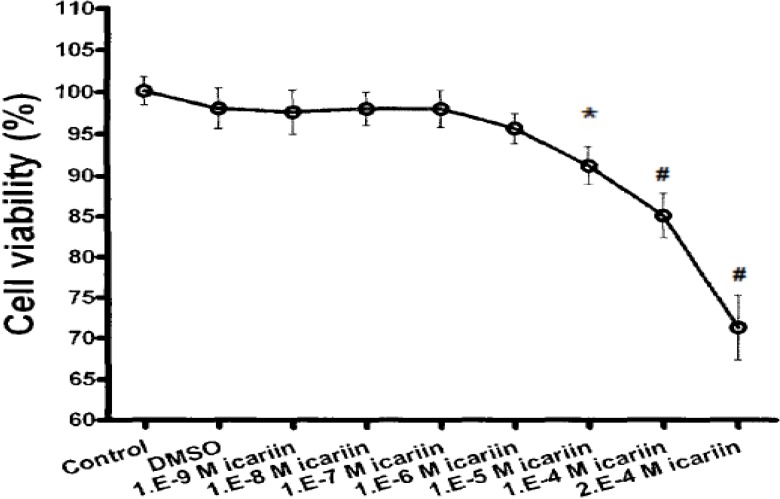
Toxicology of different concentrations of icariin toward hBMSCs (Cell viability %) (n = 6).

The hBMSC OD values were stable when treated with 10^−9^ M to 10^−6^ M icariin. This means there is no cytotoxicity when the concentration of icariin was smaller than 10^−6^ M. But the OD values decreased when the concentration of icariin was larger than 10^−5^ M (* P < 0.05, ^#^ P < 0.01). This means there is cytotoxicity when the concentration of icariin was larger than 10^−5^ M. The result showed that the cytotoxicity of icariin limited the cell viability (Figure 4).

**Figure 5:**
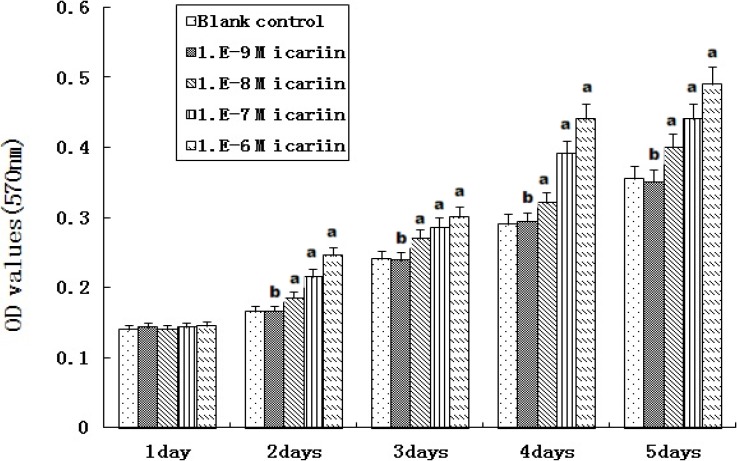
Proliferation of hBMSCs stimulated by icariin (n = 6).

### 2.5. Proliferation of hBMSCs Stimulated by Icariin

At icariin concentrations of 10^−8^ to 10^−6^ M, OD values increased substantially from days 2 to 5 and were higher than that of the blank control (^a^ P < 0.05) (Figure 5). OD values increased with increasing icariin concentration, and a dose-dependent effect was showed. On the other hand, OD values for hBMSCs treated with 10^−9^ M icariin showed no difference when compared to the blank control (^b^ P > 0.05). Icariin had a dose-dependent effect on the proliferation of human bone mesenchymal stem cells.

### 2.6. Osteogenic Differentiation of hBMSCs Stimulated by Icariin

**Figure 6:**
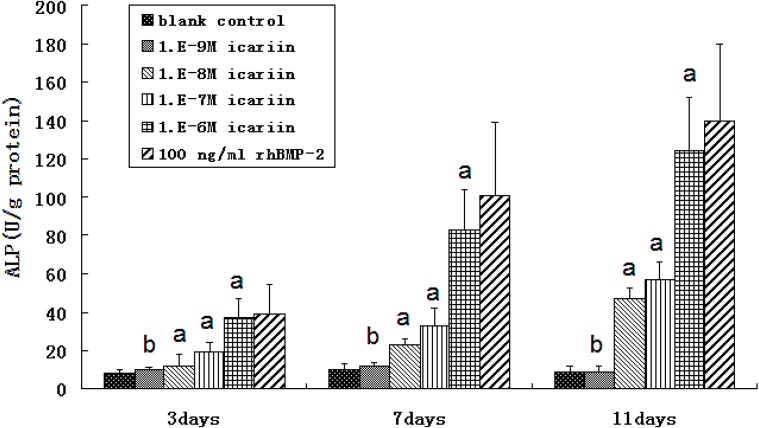
The expression of ALP (n = 6).

**Figure 7:**
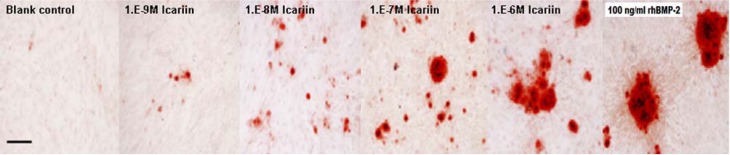
The observation of mineralized deposits (bar indicates 100 μm).

At icariin concentrations of 10^−8^ to 10^−6^ M, ALP expression increased substantially from days 3 to 11 and was higher than that of the blank control (^a^ P < 0.05). ALP expression increased with increasing icariin concentration, and a dose-dependent effect was showed. On the other hand, icariin at 10^−9^ M showed no effects on the expression of ALP from days 3 to 11 (^b^ P > 0.05). At a different time point, 10^−6^ M icariin showed the highest expression of ALP in all the icariin groups, but the expression was little lower than that of the rhBMP-2 treated group (Figure 6). Cell clones in the groups treated with 10^−6^ M icariin and rhBMP-2 were almost completely covered with mineralized deposits, whereas less bone nodules were noticed in the other icariin groups. Bone nodules increased with increasing icariin concentration and no nodule formed in the blank control (Figure 7).

In the present study, we isolated and purified hBMSCs from the bone marrow of normal volunteers. By quantitative flow cytometry, we detected that the cells were positive for selected mesenchymal markers CD29, CD44, CD71, CD105 and CD166 and negative for hematopoietic cell-surface markers CD14, CD34 and CD45. The negative expression of hematopoietic markers in this study indicates no contamination by hematopoietic cells in the hBMSC isolation [13]. For proof of pluripotency, the cells were amplified and differentiated into three cell lineages. The lines consisted of the chondrogenic, adipogenic and osteogenic phenotypes, proving that the cells used in this study were actually pluripotent stem cells.

Due to their strong ability to induce ectopic bone formation, the BMP family proteins are the most notable among the osteogenic factors reported to date [14]. However, the clinical application of BMPs has been limited due to their unstable activity (the short half-life of BMP-2 is 7 to 16 minutes *in vivo*) and high cost [6,14]. As a flavonoid compound, icariin can be easily obtained and purified from natural herbaceous plants with low-cost separation techniques [14]. Most importantly, icariin showed a great potential in the differentiation and proliferation of hBMSCs in our present study. Thus, the application of icariin should be considered as an alternative to GFs in bone tissue engineering.

Although icariin has been shown to have a therapeutic effect on the osteoporosis of animal models *in vitro* and *in vivo* [15], its feasibility as an alternative to GFs in bone tissue engineering is still undetermined. An *in vitro* study revealed that the increase in bone mass was associated with the differentiation of bone marrow stromal cells [16] and the enhanced expression of various proteins critical to bone matrix deposition [17]. Further studies demonstrated that icariin stimulates MC3T3-E1 cell proliferation, increases ALP activity, and promotes type I collagen expression [14]. However, study of the detailed molecular mechanisms by which icariin enhances the proliferation and differentiation of pre-osteoblastic cells is far from being exhausted.

## 3. Experimental

### 3.1. Isolation and Expansion of hBMSCs

The study protocol was approved by the Institutional Ethical Committee on Human Research and was performed in compliance with human studies according to the Helsinki Declaration of 1975, as revised in 1996. Informed consent was given by all the volunteers involved. Human bone marrow samples were isolated from the posterior iliac crests of three consenting healthy adult volunteers (aged 23, 25 and 26). About 10 mL of bone marrow sample was obtained from each volunteer. Samples were placed in 50-mL falcon tubes containing phosphate buffered saline (PBS, 10 mL) supplemented with 500 µg/mL heparin. After filtering through a 100-mm filter, the samples were mixed with Hanks buffer (Invitrogen, Australia) and then lymphoprep (15 mL, Lym, Aix-Shield, Norway) was gently layered (Lym/sample was 1/2) so that the column sat below the sample. The samples were then centrifuged at 400 g without acceleration or brake for 30 min at 20 °C. The bottom layer contained erythrocytes, which were aggregated and sedimented completely through Lym. Cells located at the interface between the bone marrow sample and the Lym were collected and further resuspended in Dulbecco’s modified Eagle’s medium with low glucose (DMEM-LG, 20 mL, Gibco, Invitrogen, Australia) supplemented with 10% (v/v) fetal bovine serum (FBS) (HyClone, USA), 10 U/mL penicillin G, and 10 mg/mL streptomycin (Gibco).

The cell cultures were performed by plating the cell suspension in T75 culture flasks at 37 °C in a humidified atmosphere containing 5% (v/v) carbon dioxide. The medium was unchanged for the initial 5 days and then changed every 3 days until the culture flask became confluent. When the flasks reached confluence, cells were detached using 0.25% (w/v) trypsin-EDTA (Gibco), and the cells were taken to the next passage. For morphological observation, cells of the second passage were grown on coverslips and stained using hematoxylin and eosin (H&E) staining.

### 3.2. Phenotype Identification of Cells

Cells of the second passage were used for phenotype identification. After cultures reached confluence, cells were harvested by 0.25% trypsin-EDTA and centrifuged at 400 g for 10 min. Cells were resuspended in fluorescence-activated cell sorting (FACS) buffer (PBS + 0.1% (w/v) sodium azide + 1% (w/v) bovine serum albumin) and incubated with primary unconjugated antibodies (CD14, CD29, CD34, CD44, CD45, CD71, CD105, CD166, major histocompatibility complex (MHC) class I, and MHC class II) (Becton Dickinson Biosciences, Franklin Lakes, NJ, USA) for 25 min at 20 °C. The cells were then washed and incubated with secondary antibody (goat anti-mouse immunoglobulin G phycoerythrin conjugate) for 15 min. The cells were then washed and analyzed using flow cytometry on a FACS Calibur (Becton Dickinson, Oxford, UK). Data were analyzed using FCS Express software.

### 3.3. Inducing Differentiation of Cells

For proof of pluripotency, the cells at the second passage were differentiated into three cell lineages. Chondrogenic differentiation was induced by replacing medium with serum-free, high glucose DMEM supplemented with 10 ng/mL TGF-β1, 1 nmol/mL dexamethasone, 50 mg/mL ascorbic acid 2-phosphate, 100 mg/mL sodium pyruvate, 40 mg/mL proline, and a commercial preparation of insulin transferrin selenious acid-plus (final concentration: 6.25 mg/mL insulin, 6.25 mg/mL transferrin, 6.25 mg/mL selenious acid, 5.33 mg linoleic acid, and 1.25 mg/mL bovine serum albumin). This medium was changed twice weekly. After 2 weeks, cells were fixed with 4% paraformaldehyde and matrix deposition of proteoglycans was detected using toluidine blue staining.

Osteogenic differentiation was induced by replacing medium with DMEM containing 10% FBS, 5 mg/mL ascorbic acid, 1 mmol/mL β-glycerol phosphate, and 1 nmol/mL dexamethasone. This medium was changed twice weekly. After 3 weeks, cells were fixed with 4% paraformaldehyde, and the matrix mineralization was detected using alizarin red staining.

Adipogenic differentiation was induced by replacing medium with DMEM containing 10% FBS, 0.5 mM isobutylmethyl xanthine, 100 to 200 mM indomethacin, 1 nmol/mL dexamethasone, and 10 mg/mL insulin. This medium was changed twice weekly. After four weeks, cells were fixed with 4% paraformaldehyde and stained with Oil red-O to detect the lipid droplets.

### 3.4. Cytotoxicity Test of Icariin

The dimethyl sulfoxide (DMSO) culture fluid was made by adding DMSO (1 µL) into DMEM (20 mL) containing 10% FBS. Icariin (National Institute for the Control of Pharmaceutical and Biological Products, China) was melted in the DMSO culture fluid to get different concentrations of 10^−9^ M, 10^−8^ M, 10^−7^ M, 10^−6^ M, 10^−5^ M, 10^−4^ M and 2.0 × 10^−9^ M. After phenotype identification, hBMSCs at the third passage were seeded in 96-well plates at a density of 5 × 10^3^ cells/well in DMEM containing 10% FBS for 24 h, and then cultured in the DMSO culture fluid with different concentrations of icariin. After another 24 h the level of mitochondrial activity of hBMSCs after icariin treatments was determined using a colorimetric assay that detects the conversion of 3-(4, 5-dimethylthiazolyl-2)-2,5-diphenyltetrazolium bromide (MTT, Sigma, St. Louis, MO, USA) to insoluble formazan. The absorbance was determined at 570 nm using an enzyme linked immunosorbent assay reader (Elx 800, Bio-Tek, Winooski, VT, USA). Cells in DMEM containing 10% FBS without icariin were used as a blank control. The cell viability was evaluated by comparing the OD value of icariin group with the control group.

### 3.5. Proliferation of hBMSCs Stimulated by Icariin

Based on the result of the cytotoxicity test of icariin, 10^−9^ M to 10^−6^ M icariin was used to evaluate the proliferation of hBMSCs. A series of cultures was stimulated for 1 to 5 days. During the experiment, the treatments (including medium and supplements) were changed every 2 days and fresh icariin was added at each medium change. The level of mitochondrial activity of hBMSCs after icariin treatments was determined using the above-mentioned MTT method everyday of culture. Cells in DMEM containing 10% FBS without icariin were used as a blank control. Cells stimulated with 100 ng/mL rhBMP-2 were used as a positive control.

### 3.6. Osteogenic Differentiation of hBMSCs Stimulated by Icariin

Based on the result of the cytotoxicity test of icariin, 10^−9^ M to 10^−6^ M icariin was used to evaluate the osteogenic differentiation of hBMSCs. Cells at the third passage were seeded in 96-well plates at a density of 2 × 10^7^ cells/well in DMEM containing 10% FBS without osteogenic supplements. After 24 h, icariin was added at concentrations ranging from 10^−9^ M to 10^−6^ M. Cells cultured without icariin were used as a blank control. Cells stimulated with 100 ng/mL rhBMP-2 were used as a positive control.

At days 3, 7 and 11, the cells were washed three times with PBS, obtained by scraping the culture dish and suspended in 1 mL deionized water. The cells were then completely lysed by sonication for 10 minutes with a sonic cell disruptor (Cosmo Bio, Tokyo, Japan). After sonicates were centrifuged at 400 g for 10 min, the supernatants were used for ALP activity assay. The protein concentrations were determined with the BCA protein assay reagent. ALP activity was expressed as the ratio of the reading of the total automatic biochemistry instrument (Hitachi, Tokyo, Japan) and the corresponding protein concentration (U/g).

At day 21, cells were fixed with 4% paraformaldehyde and matrix mineralization was detected using Alizarin Red-S staining. After the Alizarin Red-S solution was removed, cultures were washed with PBS for 15 min at 20 °C. Bound dye was solubilized in 10% (w/v) cetylpyridinium chloride (Aldrich Chemical, St. Louis, MO, USA) in 10 mM sodium phosphate at pH 7.0. The extracted stain was then transferred to a 96-well plate and read on an ELISA reader at a wavelength of 570 nm.

### 3.7. Statistical Analysis

Statistical analyses were performed using SPSS software, version 12.0 (SPSS Inc, Chicago, USA). The data were presented as the mean ± standard deviation and levels were compared by the One-Way ANOVA and Student’s t-test. P values less than 0.05 were considered significant.

## 4. Conclusions

These results demonstrate that icariin had a dose-dependent effect on the proliferation of hBMSCs and also could enhance the osteogenic differentiation of hBMSCs in a suitable concentrations range from 10^−9^ M to 10^−6^ M, but when greater than 10^−5^ M, the toxicity limited its use. The extremely low cost of icariin and its high abundance make it appealing for bone regeneration.
